# Smartphone-Based Cognitive Telerehabilitation: A Usability and Feasibility Study Focusing on Mild Cognitive Impairment

**DOI:** 10.3390/s24020525

**Published:** 2024-01-15

**Authors:** Caterina Formica, Mirjam Bonanno, Chiara Sorbera, Angelo Quartarone, Fabio Mauro Giambò, Angela Marra, Rocco Salvatore Calabrò

**Affiliations:** IRCCS Centro Neurolesi Bonino-Pulejo, Cda Casazza, S.S. 113, 98124 Messina, Italy; katia.formica@irccsme.it (C.F.); chiara.sorbera@irccsme.it (C.S.); angelo.quartarone@irccsme.it (A.Q.); fabio.giambo@irccsme.it (F.M.G.); angela.marra@irccsme.it (A.M.); roccos.calabro@irccsme.it (R.S.C.)

**Keywords:** telerehabilitation, smartphone, neurorehabilitation, neurodegenerative disorders

## Abstract

The implementation of cognitive health apps in patients with mild cognitive impairment (MCI) is challenging because of their cognitive, age, and other clinical characteristics. In this project, we aimed to evaluate the usability and feasibility of the Rehastart app tested in MCI patients. Eighteen subjects affected by MCI due to neurodegenerative disorders (including Parkinson’s disease, multiple sclerosis, and amnestic/multidomain MCI) and eighteen healthcare professionals were recruited to this study. Patients were registered on the app by clinicians and they were assigned a protocol of specific cognitive exercises. The recruitment was conducted in the period between March and June 2023. The trial testing of the app consisted of three sessions per week for three weeks, with each session lasting about 30 min. After three weeks, the participants as well as medical personnel were invited to rate the usability and feasibility of the Rehastart mobile application. The instruments employed to evaluate the usability and feasibility of the app were the System Usability Scale (SUS), The Intrinsic Motivation Inventory (IMI) and the Client Satisfaction Questionnaire (CSQ). We did not find statistically significant differences on the SUS (*p* = 0.07) between healthcare professionals and patients. In addition, we found promising results on subscales of the Intrinsic Motivation Inventory, suggesting high levels of interest and enjoyment when using the Rehastart app. Our study demonstrated that smartphone-based telerehabilitation could be a suitable tool for people with MCI due to neurodegenerative disorders, since the Rehastart app was easy to use and motivating for both patients and healthy people.

## 1. Introduction

Recent research suggests that the management of elderly people with neurological diseases requires a greater use of human and economic resources for the national health system (NHS) than management of elderly people without neurological diseases. In Italy, due to the aging of the population and the high morbidity rates of neurodegenerative pathologies, the ability of individuals to live independently is reduced [1]. Mild cognitive impairment (MCI) may represent the prodromal stage of dementia or be associated to other neurodegenerative disorders [2], conditions that will affect 13.4 million people by 2030 [3,4]. Although various neurodegenerative disorders, such as Parkinson’s disease (PD), dementia, and multiple sclerosis (MS), may have different underlying neuroanatomical and neuropathological mechanisms, they may share similar cognitive deficits in different phases of the pathologies. In particular, about 70% of PD patients experience cognitive impairments that impact their quality of life. Cognitive decline can sometimes be present before the onset of motor symptoms and worsen in the advanced stages of the disease [5]. Cognitive dysfunction in PD is associated with the pathological involvement of basal forebrain cholinergic and dopaminergic systems. The hallmark features are impairments in executive functions, attention, and visuospatial abilities [6]. On the other hand, cognitive impairment in MS has a prevalence of 45–70%, producing a negative impact on patients’ quality of life. This prevalence may vary based on the widespread changes in neural networks that contribute to cognitive dysfunction, and the presence of grey matter atrophy may be a warning sign of cognitive decline. The most frequently affected cognitive domains concern processing speed and episodic memory [7]. Early detection of cognitive decline has become an important factor in creating an adequate cognitive rehabilitative strategy [8,9]. Concerning neurodegenerative diseases, early and constant interventions over time, which also continue at home, can be useful for slowing down the progression of cognitive decline and therefore improving patients’ quality of life. Studies suggest that customization according to personal preferences and limitations is a prerequisite for developing computer-based technologies accessible for patients with MCI due to neurodegenerative disorders, especially for older individuals [10,11]. In the context of chronic diseases, self-care management has become an important issue. Cognitive training is the most commonly reported form of cognitive intervention in the field of rehabilitation. Patients can be involved in face-to-face sessions using tasks aimed at improving attention, memory, and language [12]. However, traditional interventions may not always be accessible to older individuals on a large scale due to the need to travel long distances to specialized treatment centers and limited public economic resources. For this reason, in recent years, there has been a growing interest in the application of innovative technology in this field. Indeed, touchscreen tablets and other smart systems are increasingly used to help patients with chronic diseases to improve cooperative collaboration with clinicians, and manage and treat disease symptoms, with a consequent improvement in their quality of life [13]. Indeed, these tools provide neurological patients with psychosocial interventions, clinical counseling, and cognitive and motor rehabilitation [14]. Telemedicine represents a method of providing healthcare services at a distance and remotely connecting health professionals and patients [15,16]. In Italy, telemedicine has mainly been used in the research field, with fewer applications in clinical practice. Several studies have provided evidence regarding the efficacy of ICT-based cognitive training when used as an adjunct therapy for recovering or improving cognitive performance [17,18,19]. Positive results have also been achieved when used in early intervention for individuals with MCI and age-related cognitive decline. Many devices and digital apps for people with MCI have been developed in recent years, particularly regarding cognitive training and support for people who live alone [14,20]. In this population, using technology is more difficult because users with cognitive impairment make more mistakes and need more time to use web platforms due to their difficulties in orientation [21]. Lauriks et al. suggested that people with mild to moderate cognitive impairment should be made familiar with simple technologies [22]. Another study correlated general levels of autonomy (daily life activities) and the ICT autonomy level with positive outcomes, defining it as a good predictor of the user’s level of autonomy in using technologies. This information could be useful in building the type of interface most suitable for different types of patients, with potential clinical implications [23]. Studies conducted in patients with mild to moderate cognitive impairments due to neurodegenerative disorders supported the effectiveness of a smartphone-based app with an older population, showing that the use of mHealth could improve cognitive abilities and allow generalization to outcomes in daily life, even in the presence of neurodegenerative disorders [24,25,26].

Given the growing interest of the scientific community in mHealth, we proposed a project investigating the usability of a smartphone-based app for cognitive rehabilitation in patients with neurodegenerative disorders. The Rehastart project (“Research and Development of technologies and methodologies in Tele-Rehabilitation (REHASTART)”) has created a model of a cognitive-oriented app for people with cognitive impairment. However, the implementation of cognitive health apps in these patients is challenging because of their age, as well as cognitive impairments and other clinical characteristics. For this reason, the key factors for demonstrating the potential usefulness of the app are its usability and feasibility. Usability is defined as the “effectiveness, efficacy and satisfaction with which specified users can achieve goals in particular environments” [27], while feasibility is the “assessment of the practicality of a proposed system” [23]. In this case study, usability and feasibility were assessed by the observation of users’ experiences with the app. Previous studies have evaluated the usability and feasibility in MCI populations of smartphone model-based apps and information and communication technology (ICT) design [28,29], demonstrating a positive correlation between levels of personal autonomy and ICT autonomy level. Moreover, a narrative review found that mobile technologies based on self-monitoring had a positive impact on the everyday life of middle and older populations with neurological disorders, and patients with depression and chronic pain, by providing cognitive, social and motor improvements and promoting digital health [16].

This study evaluated the usability and feasibility of the Rehastart app for patients with MCI due to diverse neurodegenerative disorders. These features were also investigated in a group of healthy controls.

## 2. Materials and Methods

Eighteen subjects (13 males and 5 females; mean age—56.22 ± 12.98) affected by MCI due to neurodegenerative disorders, including PD, MS and pure amnestic/multidomain MCI, and eighteen healthcare professionals (mean age—32.5 ± 7.03) were recruited for this study. We included patients who met the following inclusion criteria: (i) affected by neurodegenerative disorders (Parkinson’s disease, multiple sclerosis, or amnestic/multidomain mild cognitive impairment); (ii) had a Montreal Cognitive Assessment (MoCA) [30] score of 20 to 30 points; and (iii) had a smartphone with iOS or Android and an internet connection. Otherwise, we excluded participants with (i) specific sensory–motor disabilities (visual or hearing); (ii) patients with moderate motor impairment; and (iii) patients with language deficits. Most of the patients recruited had a high school degree (66.6%), or university degree (22.22%), and their cognitive impairment was moderate (MoCA: 25.8 ± 3.39). The healthcare professionals were physiotherapists (38.8%), psychologists (55.5%), and one physician (5.5%), with a university degree (100%) (see Table 1 for more details).

### 2.1. Study Design

This feasibility and usability study aimed to evaluate the simple use of a smartphone application (Rehastart App, Khymeia, Padova, Italy) for telerehabilitation in people with MCI due to neurodegenerative disorders. The Rehastart application is recognized as an electromedical device of class I, equipped with certification CE. Once Rehastart had been downloaded onto the patients’ personal smartphones, the level of difficulty was set according to patient characteristics as estimated based on performance (number of errors and time needed) in four time-limited (1 min) trials aimed at improving various cognitive domains (memory, attention and executive functions). Specifically, MCI patients performed a 3-week cognitive training program remotely at home using their personal smartphone, 3 times per week (each session lasting about 30 min) for a total of 9 sessions. The app reminded patients of the training through the calendar at the agreed time (09:00) three times per week (Monday, Wednesday, and Friday). The time for each cognitive domain was standardized among the participants. Every week the clinician monitored the responses of the patients through a daily report. Based on the results, clinicians changed the order of presentation of stimuli and regulated the difficulty level in order to avoid the phenomenon of habituation.

Healthcare professionals (HPs) registered the patients’ profiles on the Rehastart app and assigned the protocol of cognitive exercises (e.g., memory, attention, and executive functions tasks). Indeed, HPs instructed patients to use the app during the first session. After the trial period of 3 weeks, patients were submitted to usability and satisfaction evaluations of the smartphone app through specific questionnaires, administered via phone call. In addition, teleconsultations were carried out, when needed, to solve concerns or difficulties.

On the other hand, the HPs, who included psychologists, physiotherapists, and neurologists, were trained in the use of the Rehastart app by an informatic engineer (FMG). The HPs, as well as the MCI patients, tested the usability of the Rehastart app for 3 weeks. Then, HPs completed self-report questionnaires regarding the usability and feasibility of the Rehastart App.

The research was conducted from March to June 2023. Written informed consent was obtained from the MCI patients and the HPs. The study was conducted in accordance with the Declaration of Helsinki and approved by the Ethical and Research Committee of IRCCS Centro Neurolesi “Bonino-Pulejo”, Messina, Italy (ID: 50/2021). The study has been funded by Current Research Funds, 2023, Italian Ministry of Health.

### 2.2. Software Design for the Medical Backend

#### 2.2.1. Case Management

The application is based on two different technical sections: (i) platform managed by clinicians/physiotherapists/psychologists (Healthcare Professionals—HPs), and (ii) platform used by patients. The Rehastart application can be downloaded on smartphones and tablets from the Apple Store or the Google Play Store, through an available internet connection. In addition, this application can share data in real time thanks to the use of external devices or tools, like inertial sensors.

#### 2.2.2. Personnel Management—System Implementation for Healthcare Professionals

Personnel management refers to the creation of accounts for HPs, which are created by the clinic. Once they receive their login credentials, HPs can add patients and prescribe rehabilitation protocols for them from their Rehastart account. After the login, the HPs can view the list of their own patients (“Associated patients”) and he/she can add other patients from the tab “Invite patients”. From the HP’s profile, the user is allowed to create the patient profiles with a username and password and to add/choose the following settings:

(a) Patients’ data (such as age, sex, and pathology).
(b) Measurements (such as vital parameters, which can be added by both HPs and patients). The clinician is allowed to add new measurements, including temperature, saturation, blood pressure, nasal swabs, and monoclonal antibodies.
(c) Prescriptions.

#### 2.2.3. Exercise Management and Classification

**Figure 1:**
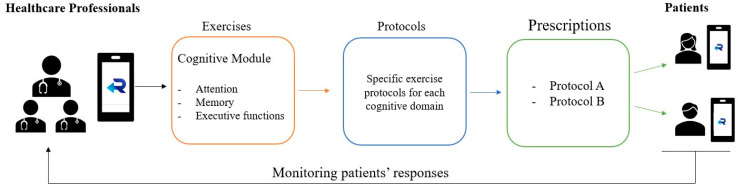
Scheme of exercise management for Rehastart application. Healthcare professionals, via their account on their smartphone, can choose exercises from a cognitive exercise database, then they create a protocol which becomes a prescription for the patients. Lastly, patients can view their exercise protocol on their smartphone app. Clinicians are then allowed to monitor patients’ responses.

The function of exercise management and classification is to show the patient’s demographic and clinical data, enable video chats with patients, and show the exercise prescription that was assigned. From the exercise management section of the app, the HP can see the exercise list and add, edit, and delete exercises. It is possible to display all clinical data if available. HPs can create an exercise protocol in which they can decide the type (motor and/or cognitive) of exercises, the number of exercises, modify the difficulty, and modify the number of series to perform and the time of each execution. Also, they can monitor the responses of the patients through a daily report (Figure 1).

### 2.3. Design of the Smartphone Application—For Patients

#### 2.3.1. Login Screen

The HP provides the patient with access credentials to the app. The patient can log in into the app by entering their username and password.

#### 2.3.2. My Protocol

After logging in, the patient can click on “My protocol” on the calendar to view their list of tasks for the day. By pressing on the specific event displayed on the calendar, the app accesses the relevant execution page, from which it is possible to proceed as specified by clicking on “START”. Then, a list of exercises is displayed and the patient can select an exercise to begin the task. For each exercise, the preview is first displayed, then the audio with instructions. At the end of the exercise, the app automatically moves on to the next exercise until the end of the protocol. Using the other buttons, patients can view the vital parameters recorded and insert new ones. Furthermore, the patient can view the list of clinicians associated with the account and send a message or make a video call with an HP.

### 2.4. Exercise Database Design

**Figure 2:**
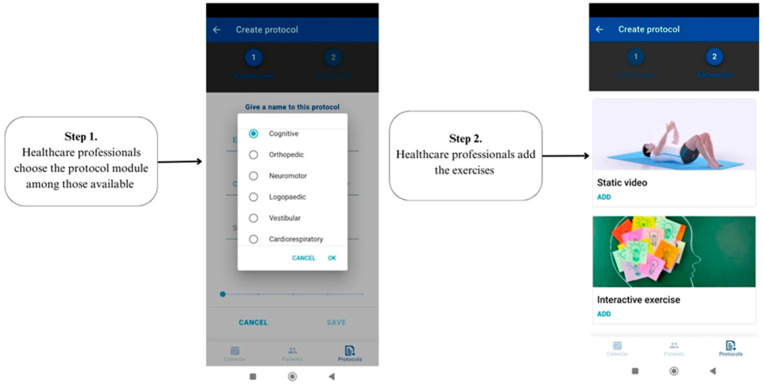
Shows both steps for the prescription of protocol exercise using the Rehastart App.

The therapeutic exercise protocols can easily be added through the HP backend server. The system can include motor tasks, stretching and range-of-motion exercises, endurance, and strength training. Also, cognitive training is available in the App, and can be carried out for a wide range of cognitive domains, such as attention, memory, visuo-spatial skills, and executive functions. Exercises were selected according to difficulty, domain, and time of execution. Cognitive exercises were interactive and were carried out by the patient using the touchscreen of their personal device, without the use of external sensors. Specifically, in our study we tested the usability of cognitive tasks presented in the app (Figure 2).

### 2.5. Outcome Measures

User acceptance and satisfaction were evaluated after the training, within one week at the latest. The motivation of the users and the usability of the system during training, experienced by both the patients and medical personnel, were measured by using the following standardized questionnaires: the Intrinsic Motivation Inventory (IMI) and the System Usability Scale (SUS), respectively. In addition, patients were also interviewed about their satisfaction with the service, using the Client Satisfaction Questionnaire (CSQ).

The IMI is a questionnaire that provides qualitative information on the content and level of motivation that patients experienced during the treatment. Each IMI item consists of a seven-point Likert scale, with responses ranging from “not at all true” to “completely true”. Higher scores mean a more positive result for motivation [31]. In addition, IMI–22 items comprise four different sub-items including interest/enjoyment (I/E), perceived competence (PC), pressure/tension (P/T), and perceived choice (PCh). The I/E subitems evaluate the self-reported intrinsic motivation, consisting of the interest and inherent pleasure experienced when the subject is performing an activity. PCh subitems evaluate whether individuals feel they engage in an activity because they choose to do it; PC measures how effective individuals feel when they are performing a task; lastly, P/T evaluates if participants feel pressure to succeed in an activity, and this is considered to be a negative predictor of intrinsic motivation. The reliability coefficient of the total IMI was found to be 0.86 using Cronbach’s alpha. Therefore, the IMI was considered by various authors [32,33,34] to be a valid tool in psychological and educational research.

The SUS is a scale that consists of 10 items and evaluates the user’s subjective usability experience. The questions are assessed on a five-point Likert scale, with responses ranging from “strongly agree” to “strongly disagree”. A high score means better usability. Scores of 90 are exceptional, whereas scores between 60 and 80 are good and promising and SUS scores lower than 50 indicate usability difficulties [35]. The psychometric properties of the SUS have been widely studied, with reported reliability scores of between 0.79 and 0.97 [36,37]. According to Mol et al. [38], the total sum score of the SUS appears to be a valid and interpretable measure for assessing the usability of internet-based interventions when used by professionals in mental healthcare.

In addition, we administered the Client Satisfaction Questionnaire (CSQ) to patients. This is a structured survey used to assess the level of satisfaction for the clients. The scores are assessed on a 4-point Likert scale ranging from 1 (low satisfaction) to 4 (high satisfaction). Scores range from 0 to 24 and higher scores indicate greater satisfaction [39]. The reliability of this questionnaire ranges from r = 0.35 to r = 0.99, depending on various factors (e.g., the context in which the questionnaires is filled out, visual presentation of the questions, and other similar factors) [40]. In addition, scores on the CSQ have been shown to correlate with treatment outcomes, measured both in symptom relief and well-being, and treatment adherence; in this sense, higher satisfaction is associated with treatment adherence [41].

### 2.6. Statistical Analysis

Statistical analyses were performed using open-source software R 4.1.1 (Vienna, Austria) for Windows. The sociodemographic data were expressed as mean and standard deviation (as reported in Table 1), while the usability outcomes (SUS, IMI and CSQ) were expressed as the median (first–third quartile), in order to describe all the results related to user acceptability. The Mann–Whitney U test (two tails, if appropriate) was used to compare the patients’ and the health professionals’ outcomes, considering *p* < 0.05 as statistically significant.

## 3. Results

### 3.1. Patients’ Results

We found that the usability scores were high among patients, as reported in Table 2.

In particular, MCI–MS as well as amnestic MCI patients reported a higher median SUS score than MCI–PD patients. The scores regarding the CSQ were quite similar among the different etiologies of MCI, reflecting high levels of satisfaction for all patients. Regarding IMI scores, we found high levels of I/E, suggesting that the smartphone-based rehabilitation was enjoyable and interesting. In addition, we found reduced levels of P/T which reflect that patients felt no pressure to perform the exercises.

Moreover, we compared SUS and IMI sub-items scores between medical personnel and patients. We did not find statistically significant differences on the SUS (*p* = 0.07), or on IMI subitems I/E (*p* = 0.14) and P/T (*p* = 0.39), between medical patients and medical personnel. On the other hand, we found statistically significant differences in PC (*p* < 0.03) and in PCh (*p* < 0.02) between patients and medical personnel (Table 3).

### 3.2. Healthcare Professionals Results

HPs had high scores in the usability outcomes on both the SUS and IMI. However, physiotherapists and physicians seemed to have higher median scores compared to psychologists, especially regarding SUS scores.

Regarding the IMI, HPs reported higher scores in I/E, PC, and PCh than P/T, which remained low among the different medical professionals. These results suggest that the Rehastart app was perceived to be useful and easy to use by HPs, without any feelings of pressure or tension during its use. In addition, HPs perceived themselves to be more confident in using the smartphone app after the trial period, as reported in Table 4.

## 4. Discussion

The aim of this study was to evaluate the usability and feasibility of the Rehastart Application for both MCI patients and HPs. We found that both HPs and patients found the app easy-to-use and motivating, as shown by the high scores obtained on the IMI and SUS.

In recent decades, there has been a growing use of apps for monitoring general health (by recording clinical parameters), as well as for motor and cognitive rehabilitation. During the COVID-19 pandemic period, the use of telemedicine has increased further [42]. 

To the best of our knowledge, this is one of the few pieces of research dealing with smartphone-based telerehabilitation in a sample of patients with neurodegenerative disorders. Indeed, the Rehastart app has been properly developed for telemotor and cognitive rehabilitation in MCI patients. The adoption of the app by older people could represent a challenge for this type of population, who are likely to be unfamiliar with ICT and digital therapeutics [43]. For example, it has emerged from the literature [44,45] that older patients have reduced experience of using technologies and prefer face-to-face interaction. This can negatively affect the outcomes related to remote home training. In fact, Eicher et al. [46] highlighted some differences between older and younger people in terms of the usability of rehabilitation systems. This means that clinicians need to be aware of the importance of selecting the right patients in order to maximize adherence to the treatment and avoid the frustration of the patients. However, after an adaptation training program on the use of technological systems, older and younger people seemed not to show substantial differences in usability and feasibility outcomes [46]. This finding is in line with our results, since HPs and patients were particularly different in terms of educational level, but this did not affect our usability results because patients were adequately trained in using the app on their smartphone.

In this study, we sought to evaluate the design and implementation of the Rehastart app, including exercise protocol creation, the dashboard for use by the HPs, and data management, and test the usability and functionality for both patients and clinicians. Despite the growing body of data on the usability of digital health applications, studies on the evaluation of HP user experience are still scarce. In this vein, our study is one of the few [47,48,49] to also evaluate the user experiences of HPs, who used a smartphone-based app for telerehabilitation for the first time. For these reasons, our findings are encouraging since we did not find statistical significance in usability outcomes (according to the SUS) between HPs and patients. This could indicate that our smartphone application for telerehabilitation was easy to use for both healthy people and neurological patients with MCI. If we also consider the sociocultural and educational differences between the two groups, this result is even more promising. In line with our findings, a recent study [24] investigated the usability of a telecognitive training app for patients with PD. The authors found that telerehabilitation via mobile app could be a promising tool, in terms of usability and patients’ perception of the achievement of “improvement of cognitive abilities”. According to a systematic review [50], measuring the usability and user experiences of cognitive intervention technologies for people with MCI or dementia provides an integrated view that can contribute to their proper development. In fact, it is not only important to know if the technology is easy to use to achieve therapeutic goals, but also whether the user perceives it as pleasant. In this sense, our results regarding intrinsic motivation in using the Rehastart app are also promising, as demonstrated by the IMI scores. Both patients and HPs reported interest and enjoyment when using it. The differences between the two groups in PC and PCh scores could indicate that the patients needed greater cognitive resources to complete the cognitive exercises due to the presence of mild deficits. These data let us assume that the overall experience with the Rehastart app was positive and satisfactory. The findings are in line with previous research into smart apps [51]. Contrary to previous expectations, older people and MCI patients with neurodegenerative diseases are able and willing to use ICT solutions. Md Fadzil et al. [52] demonstrated that older people with and/or without MCI perceive digital healthcare technology as a supportive platform for them to have better communication and provide a sense of security. However, some concerns remain related to social support, which seems to be required to improve adherence to telerehabilitation and its effectiveness.

New research in this field should focus on the development of new methods and new approaches to the assessment of the usability and feasibility of such applications. We assumed that a good approach to the validation of the usability and feasibility of our app was to consider the opinions of clinicians both on the design for medical backend management and on the creation of protocols and the accessibility of cognitive protocols for patients. In fact, our results suggest that the Rehastart app was perceived as useful and easy to use by HPs without any feelings of pressure or tension during its use. This method has proven to be effective, as we have seen no differences in the SUS scores between the patient and clinician groups. The data highlight, especially in the patient group, that age-related, sociocultural, and educational barriers to the use of this app can be reduced. It is already known that the use of smartphones or tablets is useful for memory and other cognitive domain training [24,53], but these findings do not correspond to the reality in which the perceived potential does not coincide with the actual use of these technologies. The development of apps more accessible to the elderly population, even without the support of caregivers or clinicians, could facilitate the continuation of rehabilitative treatment in an ecological way in patients with MCI [54,55]. Another pearl from the study regarding the use of remote home training via mobile apps is the low-cost implementation for the NHS in guaranteeing the continuity of healthcare assistance [56]. In fact, other telerehabilitation systems, like Virtual Reality Rehabilitation System (VRRS)-HomeKit, are more expensive [57], costing around EUR 36,000. In contrast, the Rehastart app can be downloaded free, reducing expenditure on healthcare. Furthermore, the independence in the use of the app increases self-esteem, reduces the sense of frustration, and contributes to maintaining autonomy and a better quality of life.

However, this study has some limitations to acknowledge, including the small sample size and the heterogeneity of the patient sample in terms of etiology. Another limitation that should be stated is the lack of motor training with sensors, which would positively contribute to increasing patients’ compliance. However, this was intended as a pilot study, aimed at investigating the feasibility of the new app and paving the way for multicenter studies focusing on its effectiveness.

## 5. Conclusions

Our study demonstrated that smartphone-based telerehabilitation could be a suitable tool for people with MCI due to neurodegenerative disorders, since the Rehastart app was easy to use and motivating for both patients and clinicians. However, future studies with larger and homogenous samples are needed to shed some light on the field of usability evaluation of smartphone applications. Furthermore, patients’ opinions should be considered in the development of smartphone app design to meet specific patients’ needs in terms of accessibility, user interaction and quality of feedback during exercises.

## Figures and Tables

**Table 1:** Sociodemographic characteristics of the study sample. Continuous values are expressed as mean ± standard deviation, whereas categorical values are expressed as frequencies and/or percentages.

	Patients (N = 18)	Healthcare Professionals (N = 18)	*p*-Value
Age	56.22 ± 12.98	32.5 ± 7.03	<0.01
Gender			0.09
Male	13(55.5)	8 (44.4)	
Female	5(27.7)	10 (55.6)	
Education			<0.01
Middle school	2 (11.11)	0 (0.00)	
High school	12 (66.6)	0 (0.00)	
University	4 (22.22)	18 (100.0)	
Etiology		NA	NA
MCI–PD	13 (72.22)		
MCI–MS	4 (22.22)		
MCI–AM	1 (5.55)		
MoCA	25.8 ± 3.39	NA	NA
Healthcare professionals	NA		NA
Physiotherapist		7 (38.88)	
Psychologist		10 (55.55)	
Physician		1 (5.55)	

Legend: MCI–PD (mild cognitive impairment–Parkinson’s disease), MCI–MS (mild cognitive impairment–multiple sclerosis), MCI–AM (mild cognitive impairment–amnestic multidomain), MoCA (Montreal Cognitive Assessment).

**Table 2:** Usability comparison among patients reported as median (1°–3° quartile).

Usability Outcomes	MCI-PD (N = 13)	MCI-MS (N = 4)	MCI-PA (N = 1)
SUS	88.75 (87.5–97.5)	90 (89.37–90)	90
CSQ	20.5 (20–21.75)	21 (20–21)	22
IMI			
I/E	4.85 (4.57–5.4)	4.71 (4.10–4.98)	4.85
PC	5.2 (5–6.2)	5.1 (4.65–5.45)	5
PCh	6.7 (6.45–6.8)	6.6 (6.3–6.85)	6.8
P/T	2.2 (1.3–2.6)	2.3 (1.75–2.8)	3.4

Legend: SUS—System Usability Scale; CSQ—Client Satisfaction Questionnaire; IMI—Intrinsic Motivation Inventory; I/E—interest/enjoyment; PC—perceived competence; PCh—perceived choice; P/T—pressure/tension.

**Table 3:** Statistical comparison between patients and medical personnel, calculated by two-tailed Mann–Whitney U test. Data are expressed as median (1°–3° quartile).

Usability Outcomes	Patients Median (1°–3° Quartile)	Medical Personnel Median (1°–3° Quartile)	*p*-Value
SUS	90 (87.5–90)	87.5 (72.5–90)	0.07
IMI			
I/E	4.85 (4.57–5.4)	5.57 (4.88–5.85)	0.15
PC	5.2 (5–6.2)	5.6 (5.45–6.35)	**0.03**
PCh	6.7 (6.4–6.8)	6.2 (3.7–6.4)	**0.02**
P/T	2.4 (1.4–3.2)	1.8 (1.6–2.9)	0.39

Legend: SUS—System Usability Scale; IMI—Intrinsic Motivation Inventory; I/E—interest/enjoyment; PC—perceived competence; PCh—perceived choice; PT—pressure/tension. Statistical significances are in bold.

**Table 4:** Usability comparison among Healthcare Professionals was reported as median (1°–3° quartile).

Healthcare Professionals	Usability Outcomes	Medical Personnel Median (1°–3° Quartile)
Psychologists	SUS	82.5 (70–89.3)
Psychologists	IMI	
	I/E	5.64 (4.9–5.8)
	PC	5.7 (5.6–6.3)
	PCh	6.4 (5.7–6.8)
	P/T	1.9 (1.3–2.5)
Physiotherapists	SUS	87.5 (72.5–90)
Physiotherapists	IMI	
	I/E	5 (4.42–5.85)
	PC	5.6 (5.2–6.4)
	PCh	4.4 (2.3–6.1)
	P/T	1.8 (1.6–3.2)
Neurologist	SUS	100
Physician	IMI	
	I/E	6.1
	PC	5.6
	PCh	6.2
	P/T	1.8

Legend: SUS—System Usability Scale; IMI—Intrinsic Motivation Inventory; I/E—interest/enjoyment; PC—perceived competence; PCh—perceived choice; PT—pressure/tension.

## Data Availability

Data will be available on demand, from the corresponding author. The data are not publicly available due to privacy reasons.
